# Evaluation of stakeholder engagement in participatory action research to codesign a core outcome set for routine care provided to people living with dementia

**DOI:** 10.1002/alz70860_100653

**Published:** 2025-12-23

**Authors:** Danelle Kenny, Jack Nunn, Alyssa Welch, Hanh Dao Tran, Tracy Comans

**Affiliations:** ^1^ University of Queensland, Herston, QLD, Australia; ^2^ Science for All, Melbourne, VIC, Australia; ^3^ University of Queensland, Brisbane, QLD, Australia

## Abstract

**Background:**

Dementia impacts more than 55 million people worldwide. Health systems are increasingly challenged and burdened by ineffective and inefficient monitoring and evaluation frameworks that lack capacity to define and improve quality of care provided to people living with dementia. The COM‐IC project co‐developed a core outcome set appropriate for routine dementia care provided in Australian aged care settings by involving a range of stakeholders, including people living with dementia, carers, care workers, health professionals, and policymakers.

**Method:**

We critiqued effectiveness of stakeholder engagement using mixed methods analysis of survey data and STARDIT reports combined with critical reflection. STARDIT reporting was used throughout the project to systematically collate project contributions and all stakeholders were invited to complete a dynamic learning needs survey at quarterly intervals over the project. Data from these sources were analysed, giving consideration to stakeholder sub‐groups, skill development, formal supports facilitating participation, perceptions of research influence, research experience, overall experience in the project, identified barriers and facilitators to codesign and reaching consensus, and learning and professional skill development over the project.

**Result:**

Overall, stakeholders reported a positive experience from their involvement. Over time, there were changes to the way stakeholders perceived themselves, their abilities, and their beliefs about research engagement. The STARDIT report highlighted the extent of non‐researcher engagement as well as the extent of capacity building within the project to generate and implement research findings. Barriers and facilitators to codesign were reflected, including role definitions, use of technology, and balancing time constraints.

**Conclusion:**

Codesign using participatory action research methodology can be a considered a useful approach to include people living with dementia in research. Consideration should be given to additional resourcing requirements for appropriate facilitation of equitable participation.

